# Heart Rate-Corrected QT Interval Helps Predict Mortality after Intentional Organophosphate Poisoning

**DOI:** 10.1371/journal.pone.0036576

**Published:** 2012-05-04

**Authors:** Shou-Hsuan Liu, Ja-Liang Lin, Cheng-Hao Weng, Huang-Yu Yang, Ching-Wei Hsu, Kuan-Hsing Chen, Wen-Hung Huang, Tzung-Hai Yen

**Affiliations:** Division of Clinical Toxicology, Department of Nephrology, Chang Gung Memorial Hospital and College of Medicine, Chang Gung University, Taipei, Taiwan; Ohio State University, United States of America

## Abstract

**Introduction:**

In this study, we investigated the outcomes for patients with intentional organophosphate poisoning. Previous reports indicate that in contrast to normal heart rate-corrected QT intervals (QTc), QTc prolongation might be indicative of a poor prognosis for patients exposed to organophosphates.

**Methods:**

We analyzed the records of 118 patients who were referred to Chang Gung Memorial Hospital for management of organophosphate poisoning between 2000 and 2011. Patients were grouped according to their initial QTc interval, i.e., normal (<0.44 s) or prolonged (>0.44 s). Demographic, clinical, laboratory, and mortality data were obtained for analysis.

**Results:**

The incidence of hypotension in patients with prolonged QTc intervals was higher than that in the patients with normal QTc intervals (P = 0.019). By the end of the study, 18 of 118 (15.2%) patients had died, including 3 of 75 (4.0%) patients with normal QTc intervals and 15 of 43 (34.9%) patients with prolonged QTc intervals. Using multivariate-Cox-regression analysis, we found that hypotension (OR = 10.930, 95% CI = 2.961–40.345, P = 0.000), respiratory failure (OR = 4.867, 95% CI = 1.062–22.301, P = 0.042), coma (OR = 3.482, 95% CI = 1.184–10.238, P = 0.023), and QTc prolongation (OR = 7.459, 95% CI = 2.053–27.099, P = 0.002) were significant risk factors for mortality. Furthermore, it was revealed that non-survivors not only had longer QTc interval (503.00±41.56 versus 432.71±51.21 ms, P = 0.002), but also suffered higher incidences of hypotension (83.3 versus 12.0%, P = 0.000), shortness of breath (64 versus 94.4%, P = 0.010), bronchorrhea (55 versus 94.4%, P = 0.002), bronchospasm (50.0 versus 94.4%, P = 0.000), respiratory failure (94.4 versus 43.0%, P = 0.000) and coma (66.7 versus 11.0%, P = 0.000) than survivors. Finally, Kaplan-Meier analysis demonstrated that cumulative mortality was higher among patients with prolonged QTc intervals than among those with normal QTc intervals (Log-rank test, Chi-square test = 20.36, P<0.001).

**Conclusions:**

QTc interval helps predict mortality after intentional organophosphate poisoning.

## Introduction

Accidental or intentional ingestion of pesticides or herbicides is common in Taiwan because these poisons are easily accessible [1]. In a nationwide study [2], all 4799 organophosphate poisonings reported to Taiwan's Poison Control Centers between July 1985 and December 2006 were reviewed. Most organophosphate exposures were to a single organophosphate (80.37%) and nearly all were acute (98.37%). Ingestion was the most common route of exposure (74.50%), and attempted suicide (64.72%) was the most common reason given for exposure. Most of the reported exposure occurred in adults (93.25%) and males were more commonly exposed than females (64.95%). Most patients (61.97%) received atropine and/or pralidoxime. The mortality rate for all 4799 organophosphate poisonings was 12.71% [2].

There are 3 distinct clinical syndromes after acute organophosphate poisoning [3]: (1) acute cholinergic crisis (<0.5 d) as a result of acetylcholinesterase inhibition, (2) an intermediate syndrome (0.5–7 d) that has an underlying mechanism that is still unclear, and (3) delayed neuropathy (6–21 d) that is explained by the inhibition of neuropathy-target esterase. Acute cholinergic crisis [3] includes signs and symptoms resulting from hyperstimulation of muscarinic receptors (e.g., bradycardia, bronchoconstriction, bronchorrhea, hypotension, increased gastrointestinal motility, abdominal cramps, miosis, hypersalivation), nicotinic receptors (e.g., hypertension, tachycardia, fibrillation, fasciculation, necrosis of striated muscles), and both central muscarinic and nicotinic receptors (e.g., tremor, movement incoordination, seizures, central depression of respiration, coma, death). The intermediate syndrome [4] is characterized by the onset of proximal muscle weakness and cranial nerve palsies. Difficulty in breathing may progress to respiratory failure due to paralysis of the diaphragm and other muscles of respiration. Delayed polyneuropathy [4] predominantly affects the long nerves or tracts in the nervous system, causing symmetrical weakness of peripheral muscles in the hands and feet, and resulting in variable degrees of sensory impairment.

The cardiac complications associated with organophosphate poisoning are not fully appreciated by many medical practitioners. Organophosphate poisoning may precipitate complex ventricular arrhythmias, a frequently overlooked and potentially lethal aspect of this condition [5]. The mechanism by which organophosphates induce cardiotoxicity is still uncertain. In 1982, Ludomirsky et al [6] described 3 phases of cardiac toxicity after organophosphate poisoning: phase 1, a brief period of increased sympathetic tone; phase 2, a prolonged period of parasympathetic activity; and phase 3, in which QT prolongation is followed by torsade de pointes ventricular tachycardia and ventricular fibrillation [6]. The long QT-interval syndrome is believed to originate from intense and unequal sympathetic stimulation of myocardial fibers. QT-interval prolongation has been observed in some cases of severe bradycardia or disease of the central nervous system. Therefore, both sympathetic and parasympathetic overactivity may cause QT-interval prolongation, and it is not surprising to find QT-interval prolongation in cases of severe organophosphate poisoning.

In a preliminary study at Chang Gung Memorial Hospital [7], Chuang et al reported that patients with QTc prolongation had a higher mortality rate (19.6% vs. 4.8%, P<0.001) and a higher incidence of respiratory failure (56.7% vs. 20.6%, P<0.001) than patients without QTc prolongation. However, the data was not examined using Cox proportional hazard models, so analysis of multiple risk factors was not possible. Therefore, in this study, we investigated the clinical features, QTc interval, physiological markers, and clinical outcomes of Taiwanese patients after intentional organophosphate poisoning. We determined what association, if any, exists between these variables. Most importantly, we evaluated QTc interval prolongation as a predictor of mortality after organophosphate poisoning.

## Materials and Methods

This retrospective observational study complied with the guidelines of the Declaration of Helsinki and approved by the Medical Ethics Committee of Chang Gung Memorial Hospital, a tertiary referral center located in the northern part of Taiwan. Since this study involved retrospective review of existing data, the Institutional Review Board approval was obtained, but without specific informed consent from patients. However, the informed consent of risk of acute organophosphate poisoning and all treatment modalities (including cardiopulmonary cerebral resuscitation, etc) were obtained from all patients on their initial admission. In addition, all individual information was securely protected (by delinking identifying information from main data set) and available to investigators only. Furthermore, all the data were analyzed anonymously. On the other hand, if this study involved retrospective review of existing data plus retrospective analysis of remaining biological samples, both Institutional Review Board approval and specific informed consent must be obtained from all patients. The Institutional Review Board of Chang Gung Memorial Hospital has specifically waived the need for consent. Finally, all primary data were collected according to strengthening the reporting of observational studies in epidemiology guidelines.

### Patients

We analyzed the records of 118 patients with intentional organophosphate poisoning who were examined at Chang Gung Memorial Hospital between 2000 and 2011. Diagnosis of organophosphate poisoning was based on history of exposure, clinical effects, and serum cholinesterase activity. Blood cholinesterase activity was determined using an enzymatic method (normal values, 7–19 U/mL) immediately after patient arrival at the hospital. The pesticide involved was determined by history, container label, or product information provided by the patient. A complete clinical profile of each patient was recorded using a standardized form. We obtained the following data for each patient: age, sex, blood pressure, heart rate, type and toxicity classification of organophosphate, underlying diseases, smoking habits, alcohol consumption, use of medications that might be associated with QTc-prolongation, time elapsed between poisoning and arrival at the hospital, duration of follow-up, clinical manifestations, electrocardiogram results, blood cholinesterase level, hemogram, biochemistry, detoxification protocol used, and mortality. The electrocardiogram recordings were obtained upon arrival at the emergency department. Of the 118 patients, there were 75 patients with normal (<0.44 s) QTc intervals, and 43 patients with prolonged (>0.44 s) QTc intervals.

### Inclusion and exclusion criteria

All patients older than 18 years of age who were diagnosed with organophosphate poisoning at Chang Gung Memorial Hospital between 2000 and 2011 were eligible for inclusion in this study. Patients were excluded from this study if they were younger than 18 years of age or did not have low blood cholinesterase levels despite suspicions of exposure.

### Detoxification protocols

The protocols used to treat patients included gastric lavage with large amounts of normal saline, followed by infusion of 1 g/kg activated charcoal and 250 mL magnesium citrate via nasogastric tube. Magnesium citrate was used to prevent constipation after charcoal administration. Patients were also treated with specific antidotes that included atropine and oximes. Intravenous atropine was administered at a starting dose of 2 mg every 1–2 h, and dosing was titrated to the clearing of respiratory secretions and cessation of bronchoconstriction. Pralidoxime therapy (1 g every 4 h, intravenous) was also given to all patients with evidence of cholinergic toxicity.

### Definition of clinical events

Classification of organophosphate toxicity followed formal World Health Organization recommendations: Ia, extremely hazardous; Ib, highly hazardous; II, moderately hazardous; III, slightly hazardous; and U, unlikely to present acute hazard [8]. Acute renal failure was diagnosed if serum creatinine level increased to greater than 1.27 mg/dL in males or 1.03 mg/dL in females. Acute respiratory failure was defined as a condition of respiratory insufficiency requiring intubation and mechanical ventilation for more than 24 h, regardless of the fraction of inspired oxygen [9]. Hypotension was defined as a systolic blood pressure of less than 90 mmHg [10]. Structural heart disease was defined as non-coronary cardiovascular disease processes and related interventions [11]. Uses of medications that might be associated with QTc-prolongation were recorded. In this regard, the anti-arrhythmic agents included class Ia, class Ic, and class III agents [12]. The anti-microbials include macrolides/ketolides, certain fluoroquinolones, certain anti-malarials, pentamidine and azole anti-fungals [13]. The anti-psychotics and antidepressants included a) typical anti-psychotics such as chlorpromazine, pimozide, thioridazine, perphenazine, trifluoperazine, haloperidol, and droperidol; b) atypical anti-psychotics such clozapine, quetiapine, risperidone, sultopride, ziprasidone, and loxapine; c) tricyclic anti-depressants such as amitryptyline, amoxapine, clomipramine, desipramine, citalopram, doxepin, imipramine, nortryptyline, and trimipramine; and d) other anti-depressants such as fluoxetine, sertraline, and venlafaxine [14].

### Statistical analysis

Continuous variables are expressed as means and standard deviations and categorical variables as numbers with percentages in brackets. All data were tested for normality of distribution and equality of standard deviations before analysis. For comparisons between patient groups, we used Student's *t* test for quantitative variables and Chi-square or Fisher's exact tests for categorical variables. Mortality data were compared using the Kaplan-Meier method and significance was tested using a log-rank test. An initial univariate Cox regression analysis was performed to compare the frequency of possible risk factors associated with mortality. To control for possible confounding factors, a multivariate Cox regression analysis (stepwise backward approach) was performed to analyze those factors that were significant in univariate models (P<0.05) and met the assumptions of a proportional hazard model. We considered results that rejected the null hypothesis with 95% confidence to be significant. All analyses were performed using SPSS 12.0 for Windows (SPSS Inc., Chicago, Illinois, USA).

## Results

### Baseline characteristics

Table 1 shows the baseline characteristics of the 118 patients with organophosphate poisoning, grouped according to QTc interval. There were no significant differences in baseline variables between groups.

**Table 1:** Baseline characteristics of patients with organophosphate poisoning (N = 118).

Variable	Normal QTc (N = 75)	Prolonged QTc (N = 43)	P
QT interval, ms	323.26±42.32	390.65±51.15	0.000
QTc interval, ms	411.13±16.76	500.35±35.52	0.000
Age, years	52.04±16.94	56.37±14.65	0.163
Male, n (%)	52 (69.3)	25 (58.1)	0.219
Organophosphate type, n (%)			0.466
Mevinphos	6 (8.0)	4 (9.3)	
Parathion	3 (4.0)	1 (2.3)	
Phorate	1 (1.3)	0 (0.0)	
Terbufos	1 (1.3)	1 (2.3)	
Dichlorvos	1 (1.3)	0 (0.0)	
Fenamiphos	1 (1.3)	0 (0.0)	
Methamidophos	10 (13.3)	4 (9.3)	
Chlorpyrifos	31 (41.3)	17 (39.5)	
Dimethoate	3 (4.0)	6 (14.0)	
Profenofos	8 (10.7)	8 (18.6)	
Trichlorfon	3 (4.0)	0 (0.0)	
Acephate	2 (2.7)	1 (2.3)	
Malathion	5 (6.7)	1 (2.3)	
Organophosphate toxicity, n (%)			0.509
Ia Extremely hazardous	11 (14.7)	6 (14.0)	
Ib Highly hazardous	12 (16.0)	4 (9.3)	
II Moderately hazardous	45 (60.0)	31 (72.1)	
III Slightly hazardous	7 (9.3)	2 (4.7)	
U Unlikely to present acute hazard	0 (0)	0 (0)	
Hypertension, n (%)	14 (18.7)	7 (16.3)	0.744
Old stroke, n (%)	7 (9.3)	3 (7.0)	0.658
Coronary artery disease, n (%)	1 (1.3)	2 (4.7)	0.270
Structural heart disease, n (%)	5 (6.7)	6 (14.0)	0.190
Chronic obstructive pulmonary disease, n (%)	4 (5.3)	5 (11.6)	0.215
Malignancy, n (%)	1 (1.3)	2 (4.7)	0.270
Mental disorder, n (%)	34 (45.3)	17 (39.5)	0.541
Smoking habit, n (%)	31 (41.3)	21 (48.8)	0.429
Alcohol consumption, n (%)	30 (40.0)	19 (44.2)	0.657
Use of medications that might be associated with QTc-prolongation			
Anti-arrhythmic agents, n (%)	13 (17.63)	7 (16.3)	0.883
Anti-psychotics and anti-depressants, n (%)	20 (26.7)	10 (23.3)	0.682
Anti-microbials, n (%)	6 (8.0)	4 (9.3)	0.807
Time from poisoning to hospital, hour	6.29±14.05	4.48±4.70	0.413
Duration of follow-up, months	18.43±33.21	7.76±21.27	0.061

Respiratory symptoms were common after organophosphate poisoning (Table 2). Further, there were more cases of hypotension among these patients than among those with normal QTc interval (P = 0.019). Patients with prolonged QTc interval had lower systolic (P = 0.000) and diastolic (P = 0.001) blood pressure than patients with normal QTc interval. No other clinical (Table 2) or laboratory (Table 3) variables differed between groups.

**Table 2:** Clinical manifestations of patients with organophosphate poisoning (N = 118).

Variable	Normal QTc (N = 75)	Prolonged QTc (N = 43)	P
1. Cholinergic crisis			
Cardiovascular system:			
Systolic blood pressure, mmHg	122.40±18.54	107.05±27.23	0.000
Diastolic blood pressure, mmHg	84.01±14.57	72.12±21.47	0.001
Heart rate, beats per minute	77.15±9.40	85.28±41.77	0.108
Hypotension, n (%)	12 (16.0)	15 (34.9)	0.019
Gastrointestinal system:			
Diarrhea, n (%)	21 (28.0)	14 (32.6)	0.602
Emesis, n (%)	22 (29.3)	20 (46.5)	0.061
Respiratory system:			
Shortness of breath	47 (62.7)	34 (79.1)	0.065
Bronchorrhea	41 (54.7)	31 (72.1)	0.062
Bronchospasm	38 (50.7)	29 (67.4)	0.077
Respiratory failure, n (%)	34 (45.3)	26 (60.5)	0.114
Genitourinary system:			
Acute renal failure, n (%)	5 (6.7)	6 (14.0)	0.190
Central nervous system:			
Seizure, n (%)	6 (8.0)	4 (9.3)	0.807
Coma, n (%)	11 (14.7)	12 (27.9)	0.081
2. Intermediate syndrome, n (%)	6 (8.0)	4 (9.3)	0.807
3. Delay neuropathy, n (%)	1 (1.3)	2 (4.7)	0.270

**Table 3:** Laboratory analysis of blood from patients with organophosphate poisoning (N = 118).

Variable	Normal QTc (N = 75)	Prolonged QTc (N = 43)	P
Hemoglobin, g/dL	14.24±2.06	14.34±1.63	0.770
Blood urea nitrogen, mg/dL	17.07±20.12	15.89±6.09	0.775
Creatinine, mg/dL	1.06±0.83	1.02±0.40	0.811
Sodium, mEq/L	140.88±3.11	141.21±4.31	0.644
Potassium, mEq/L	3.60±0.54	3.41±0.49	0.071
Calcium, mg/dL	8.78±0.60	8.53±0.57	0.342
Cholinesterase, initial, U/mL	3.58±3.37	2.90±3.59	0.299
Cholinesterase, lowest, U/mL	2.95±2.90	2.35±3.20	0.298
C-reactive protein, mg/dL	9.30±20.31	6.17±5.90	0.700
Amylase, U/L	147.62±126.05	276.18±245.99	0.113
Lipase, U/L	117.00±95.11	105.00±60.03	0.780
CK-MB, ng/mL	11.37±6.75	28.89±60.65	0.376
Troponin-I, ng/mL	0.09±0.11	0.06±0.09	0.411

As shown in Table 4, most patients were treated aggressively with gastric lavage of large amounts of normal saline, followed by an infusion of 1 g/kg activated charcoal and 250 mL magnesium citrate. In addition, nearly all patients were treated with systemic atropine and pralidoxime injections. There was no difference in treatment modality between groups.

**Table 4:** Detoxification protocol and outcome for patients with organophosphate poisoning (N = 118).

Variable	Normal QTc (N = 75)	Prolonged QTc (N = 43)	P
Gastric lavage, n (%)	58 (77.3)	37 (86.0)	0.250
Active charcoal and magnesium citrate, n (%)	55 (73.3)	33 (76.7)	0.682
Atropine, n (%)	74 (98.7)	42 (97.7)	0.688
Pralidoxime, n (%)	73 (97.3)	42 (97.7)	0.910
Mortality, n (%)	3 (4.0)	15 (34.9)	0.000
Causes of mortality, n (%)			0.383
Cardiac arrhythmia	1 (33.3)	9 (60.0)	
Respiratory failure	1 (33.3)	5 (33.3)	
Sepsis	1 (33.3)	1 (6.7)	

**Figure 1:**
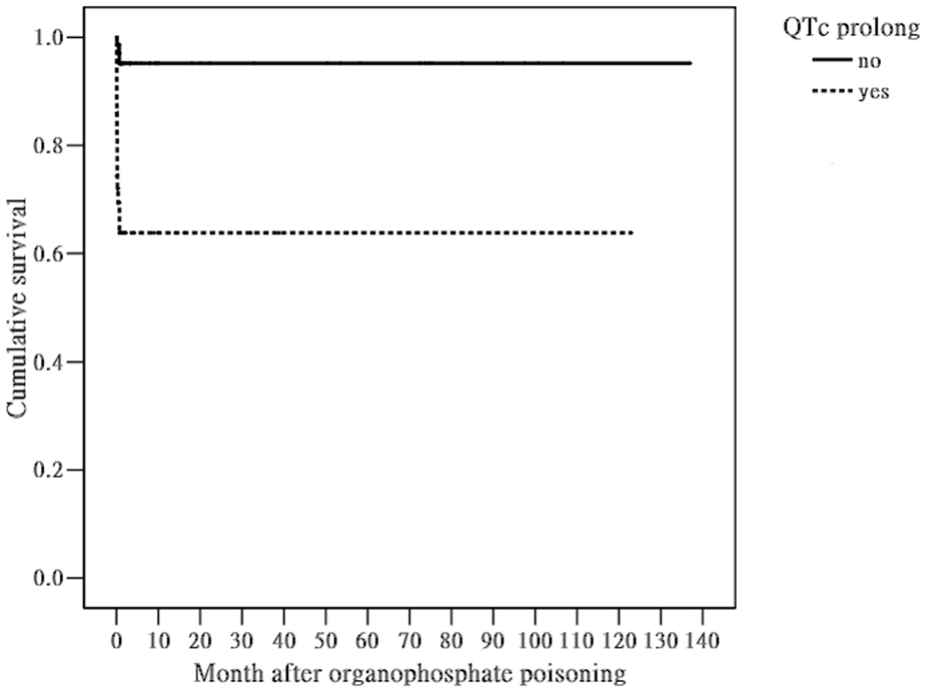
Kaplan-Meier analysis of data from patients with organophosphate poisoning, grouped according to the duration of the QTc interval. Patients with prolonged QTc intervals suffered higher cumulative mortality than patients with normal QTc intervals (Log-rank test, Chi-square test = 20.36, P<0.001).

By the end of this study, 18 of 118 (15.2%) patients had died, including 3 of 75 (4.0%) patients with normal QTc interval and 15 of 43 (34.9%) patients with prolonged QTc interval. Regarding the causes of mortality (Table 4), one patient from normal QTc interval died of cardiac arrhythmia, one died of respiratory failure, and one died of sepsis. On the other hand, 9 patients from prolonged QTc interval died of cardiac arrhythmia, 5 died of respiratory failure, and one died of sepsis. This difference was not significant (P = 0.383), probably due to the small sample size. Multivariate Cox regression analysis revealed that hypotension (odds ratio (OR) = 10.930, 95% confidence interval (CI) = 2.961–40.345, P = 0.000), respiratory failure (OR = 4.867, 95% CI = 1.062–22.301, P = 0.042), coma (OR = 3.482, 95% CI = 1.184–10.238, P = 0.023), and QTc prolongation (OR = 7.459, 95% CI = 2.053–27.099, P = 0.002) were significant risk factors for mortality (Table 5). Finally, Kaplan-Meier analysis demonstrated that cumulative mortality was higher among patients with prolonged QTc intervals than among those with normal QTc intervals (Figure 1, Log-rank test, Chi-square test = 20.36, P<0.001).

**Table 5:** Univariate and multivariate Cox regression analysis for mortality (N = 118).

Variable	Univariate analysis OR (95% CI)	P	Multivariate analysis OR (95% CI)	P
Hypotension	21.596 (6.237–74.780)	0.000	10.930 (2.961–40.345)	0.000
Respiratory failure	8.504 (1.955–36.997)	0.004	4.867 (1.062–22.301)	0.042
Coma	10.317 (3.857–27.595)	0.000	3.482 (1.184–10.238)	0.023
QTc prolongation	9.974 (2.885–34.478)	0.000	7.459 (2.053–27.099)	0.002

Note: OR odds ratio, CI confidence interval.

A direct comparison between non-survivors and survivors (Table S1) revealed that non-survivors not only had longer QTc interval (503.00±41.56 versus 432.71±51.21 ms, P = 0.002), but also suffered higher incidences of hypotension (83.3 versus 12.0%, P = 0.000), shortness of breath (64 versus 94.4%, P = 0.010), bronchorrhea (55 versus 94.4%, P = 0.002), bronchospasm (50.0 versus 94.4%, P = 0.000), respiratory failure (94.4 versus 43.0%, P = 0.000) and coma (66.7 versus 11.0%, P = 0.000) than survivors. Furthermore, the systolic (P = 0.000) and diastolic (P = 0.000) blood pressure was lower, but heart rate (P = 0.005) was higher in non-survivors than survivors.

## Discussion

It has been well demonstrated that QT-interval prolongation affects mortality rate in the general population [15]. QT-interval prolongation also affects the mortality rates of patients with a variety of diseases, including end-stage renal disease [16], coronary artery disease, congestive heart failure [17], diabetes mellitus [18], acute ischemic stroke [19], chronic liver disease [20], and chronic obstructive pulmonary disease [21]. Using a multivariate Cox regression model, we have demonstrated in this study that QTc interval prolongation might be an important risk factor for mortality after intentional organophosphate poisoning.

The relationship between QTc interval prolongation and subsequent mortality after organophosphate poisoning remains uncertain (Table 6). Baydin et al [22] reported that 35.4% of the 20 Turkish patients studied presented with prolonged QTc intervals. Further, there was a negative correlation between QTc interval and blood cholinesterase level. In a study of 13 Indian patients who died of organophosphate poisoning, Annad et al [23] found 4 with episodic tachycardia and ST-T changes, 3 with QT prolongation, and 2 with episodic bradycardia. On autopsy, all 13 patients had myocardial interstitial edema and vascular congestion, while 8 had patchy interstitial inflammation, 2 had patchy myocarditis, and 6 had a mural thrombus. Yurumez et al [24] analyzed the records of 85 Turkish patients with organophosphate poisoning and found that 47 patients (55.5%) had a prolonged QTc interval. Only 2 patients died in this study (2.4%), and no QTc prolongation was found in either patient (QTc intervals: 0.44 s and 0.40 s). The authors therefore concluded that QTc interval prolongation could not be used as a unique predictive factor in determining short-term mortality in their study [24]. In contrast, Shadnia et al [25] reported that the mortality rate of Iranian patients with long QTc intervals was significantly higher than that of those with normal QTc intervals. In a subsequent study, Akdur et al [26] found that 26 of 54 (53.7%) Turkish patients had prolonged QTc intervals. However, no significant correlation was found between poisoning severity and QTc interval. More recently [27], Vijayakumar evaluated 20 Indian patients with organophosphate poisoning and discovered that 12 patients (60.0%) had prolonged QTc intervals. However, the predictive power of QTc interval prolongation on subsequent mortality was not explored.

**Table 6:** Summary of published studies reporting a possible association between QTc and mortality rate after organophosphate poisoning.

Year	Study	Area	Sample size, n	Mortality rate, n (%)	QTc prolongation rate, %	Statistical association between QTc and mortality rate
1996	[7]	Taiwan	223	25 (11.2)	43.5	Yes
2007	[22]	Turkey	20	2 (10)	35.4	
2009	[23]	India	36	13 (36.1)	23.1	
2009	[24]	Turkey	85	2 (2.4)	55.5	No
2009	[25]	Iran	42	15 (37.5)	59.5	Yes
2010	[26]	Turkey	54	3 (5.6)	48.1	No
2011	[27]	India	20		60.0	
2012	Current study	Taiwan	118	18 (15.2)	36.4	Yes

In this study, we found that patients with QTc-interval prolongation were slightly older than those with normal QTc intervals despite not reaching the level of statistical significance (56.37±14.65 versus 52.04±16.94, P = 0.163). In a longitudinal study [28], Su et al reported that QTc interval increased significantly with age in a population of healthy elderly subjects. Elderly hearts tend to have relative midmyocardial myocyte hypertrophy and a distinct increase in connective tissue as compared to younger hearts [29]. Myocyte hypertrophy may be associated with a significant prolongation of the transmembrane action potential, explaining the prolongation of the QTc interval [30] with age. There are also reports of an exaggerated shift towards sympathetic activity in the elderly [31], and such sympathetic overactivity might be an important factor in the prolongation of the QT interval. Therefore, it is not surprising to find longer QT intervals among elderly patients with organophosphate poisoning than among younger organophosphate poisoning patients.

As shown in Table 1 and 2, there was a trend that the patients in the group with prolonged QTc were older (P = 0.163). It is possible that the increased mortality in this group might due to the age and or age-related diseases like chronic obstructive pulmonary disease and coronary artery disease. The data in the present study also showed a trend of higher percentage of patients with chronic obstructive pulmonary disease (P = 0.215), structural heart disease (P = 0.190), and coronary artery disease (P = 0.270) in the group with prolonged QTc although not reaching the level of statistical significance due to the small numbers. This group of patient also had a trend of increased frequencies in developing hypotension (P = 0.019), and respiratory distress like shortness of breath (P = 0.065), bronchorrhea (P = 0.062), bronchospasm (P = 0.077), and respiratory failure (P = 0.114). The patients in prolonged QTc group also had a trend of increased renal failure (P = 0.190) as well. Nevertheless, a direct comparison between non-survivors and survivors (Supporting Information Table S1) revealed that non-survivors not only had longer QTc interval (P = 0.002), but also suffered higher incidences of hypotension (P = 0.000), shortness of breath (P = 0.010), bronchorrhea (P = 0.002), bronchospasm (P = 0.000), respiratory failure (P = 0.000) and coma (P = 0.000) than survivors. Therefore, further large-scale study is necessary to delineate the associations between the clinical features, QTc interval, physiological markers, and clinical outcomes of patients with organophosphate poisoning.

Finally, multivariate Cox regression analysis confirmed that hypotension (P = 0.000), respiratory failure (P = 0.042), coma (P = 0.023), and QTc prolongation (P = 0.002) were significant risk factors for mortality after organophosphate poisoning. The associations between both hypotension [32] and coma with mortality are not surprising, because both are important vital signs, irrespective of the cause of disease. Similarly, it is not surprising to find an association between respiratory failure and mortality because respiratory failure is a prominent feature of acute organophosphate poisoning with an early central apnea followed by later pulmonary effects [33]. Laboratory studies also support the idea that organophosphate-induced respiratory failure results from local effects of organophosphates acting on brainstem circuits underlying respiratory rhythmogenesis, and on lung tissues underlying pulmonary secretory, airway and vascular function [33]. Furthermore, Chuang et al [7] also confirmed in their study that patients with QTc prolongation had a higher mortality rate and a higher incidence of respiratory failure than patients without QTc prolongation.

In summary, QTc interval helps predict mortality after intentional organophosphate poisoning. However, the retrospective nature of the study, small patient population, absence of routine serum magnesium measurement, and lack of serial electrocardiogram recordings limit the certainty of our conclusions.

## Supporting Information

Table S1 Comparison of clinical and physiological variables between survivors and non-survivors after intentional organophosphate poisoning (N = 118).
(TIF)
